# Supplementary material for: Association between oral microbiome and seven types of cancers in East Asian population: a two-sample Mendelian randomization analysis
Source: Front Mol Biosci. 2023 Nov 21;10:1327893. doi: 10.3389/fmolb.2023.1327893 (PMC10702768; doi:10.3389/fmolb.2023.1327893)
Supplement: Supplementary file 1 [file DataSheet1.ZIP › Supplementary Materials/MR plots of saliva/saliva═╝/colorectal cancer/pheno.2644_to_colorectal cancer_leave_one_out.pdf]

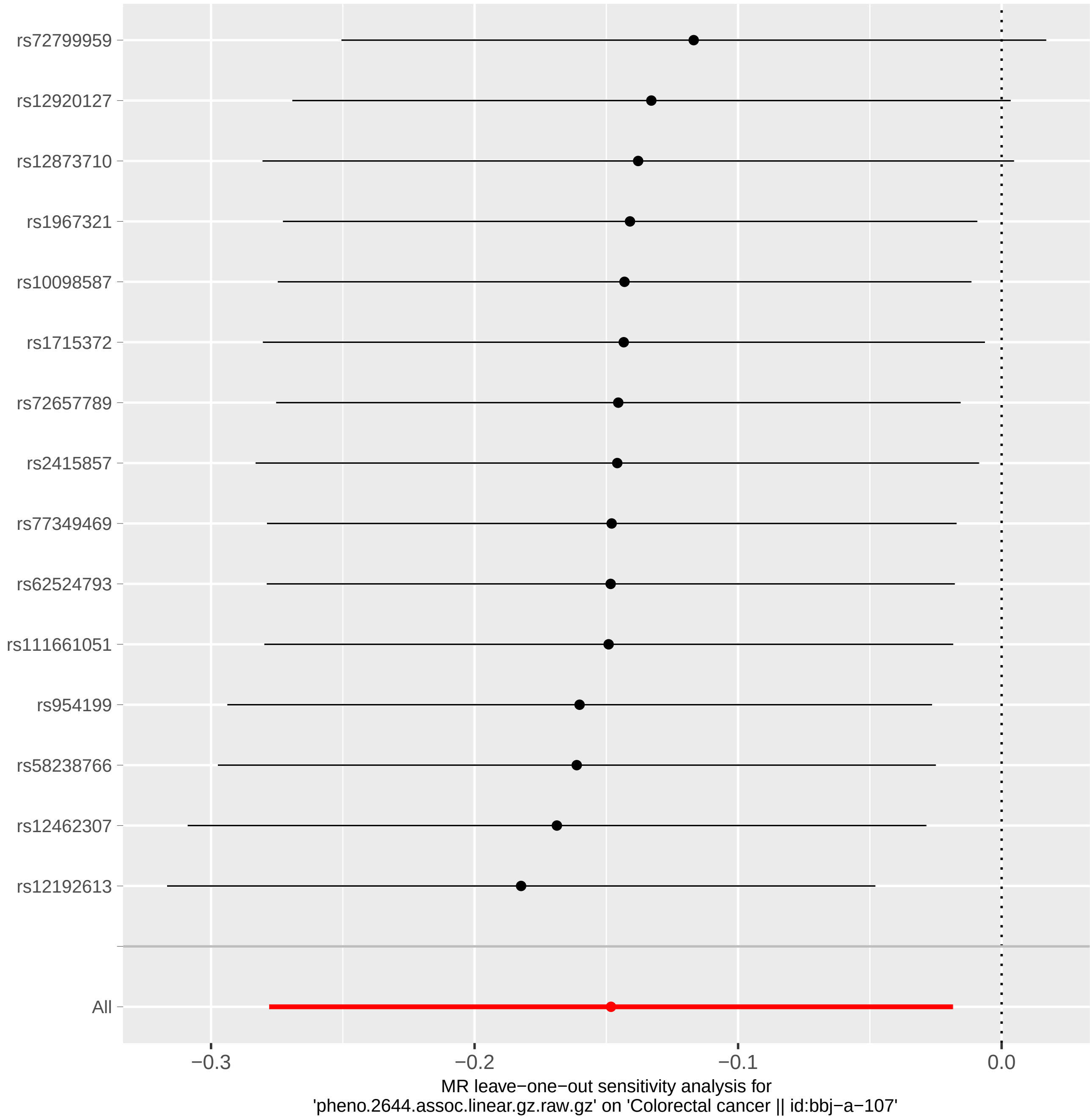
rs72799959
rs12920127
rs12873710
rs1967321
rs10098587
rs1715372
rs72657789
rs2415857
rs77349469
rs62524793
rs111661051
rs954199
rs58238766
rs12462307
rs12192613
All
−0.3
−0.2
−0.1
0.0
MR leave−one−out sensitivity analysis for
'pheno.2644.assoc.linear.gz.raw.gz' on 'Colorectal cancer || id:bbj−a−107'
